# Design of novel temperature-resistant and salt-tolerant acrylamide-based copolymers by aqueous dispersion polymerization

**DOI:** 10.1080/15685551.2022.2111845

**Published:** 2022-08-11

**Authors:** Xiang Li, Ye Ju, Yongkang Jia, Fenggang Liu, Guangpu Liu, Shuo Wang, Haoyi Wang, Shihua Mao, Jintao Yang, Guangyan Du

**Affiliations:** a China Oilfield Services Limited, Tianjin P. R.China; b College of Materials Science& Engineering, Zhejiang University of Technology, Hangzhou P. R. China

**Keywords:** Dispersion polymerization, polyacrylamide, zwitterionic structure, hydrophobic monomer, viscosity

## Abstract

Development of polymer-based flooding technology to improve oil recovery efficiency, water dispersion copolymerization of acrylamide, cationic monomer methacryloxyethyltrimethyl ammonium chloride (METAC), and anionic monomer acrylic acid (AA) were carried out in aqueous ammonium sulfate solution with polyvinyl pyrrolidone (PVP) as the stabilizer. The copolymers were characterized by ^1^H-NMR, FT-IR, TG, and SEM to confirm that they were prepared successfully and exhibited excellent salt-resistant property. Moreover, the effect of the aqueous solution of ammonium sulfate (AS) concentration, stabilizer concentration, and initiator concentration on the viscosity and size were systematically investigated. To further improve the thermal endurance properties of copolymer, hydrophobic monomers with different alkyl chain lengths were added to the above system. The acrylamide-based quadripolymer possessed prominent thermal and salt endurance properties by utilizing the advantages of zwitterionic structure and hydrophobic monomer. With the temperature rising, the viscosity retention could reach 70.2% in the water and 63.8% in the saline. This work had expected to provide a new strategy to design polymers with excellent salinity tolerance and thermal-resistance performances.

## Introduction

As most oilfields enter the mid-to-late stage of exploitation, conventional profile control and displacement technology have been severely restricted, mainly because of the reservoir heterogeneity, complex distribution of fracture pores and decrease of remaining oil saturation in the near-wellbore zone, and so on [1–6]. Improving oil recovery efficiency become an urgent problem in oil fields, currently, polyacrylamide as a water-soluble polymer has been outstandingly employed, which could improve the viscosity of water and promote oil flow ratio, further enhancing oil recovery in oilfields [7–10]. However, polyacrylamide had some own shortcomings that limited the application of oil recovery, such as salt crimping [11], high-temperature degradation [12] and shear thinning [13], which seriously restricted the oil recovery efficiency in the conditions of high temperature and high salt concentrations. At present, these problems have become the bottleneck of polymer flooding technology in oil reservoirs [14,15], thus, it is urgent to research and develop novel polymers with good temperature-/salt- resistant properties.

Zwitterionic polymers possessed equal numbers of cationic and anionic groups that had a wide application in medical material, self-cleaning surface and oil recovery [16–18]. Due to existence of both positive charges and negative charges, the typical zwitterionic polymer obtained an anti-polyelectrolyte effect – the polymer chains could stretch in salt solutions but collapse in water [19–21]. For example, Xiao et al. [22] explored the structural evidences of zwitterionic polymers with anti-polyelectrolyte effect. Their result showed that zwitterionic polymers with short carbon spacer lengths enabled to exhibit an enhanced anti-polyelectrolyte effect in salt solutions. Moreover, Dai et al. [23] also synthesized a novel amphoteric polyacrylamide (PASD) by cationic monomers (acryloxyethyl trimethylammonium chloride, DAC) and anionic monomer (sodium styrene sulfonate, SSS), which showed the excellent thermal-resistance and shear-stable property even in the concentrated salt solution. These research all found that both the solution viscosity and polymer solubility was increased at the higher salt concentrations, mainly deriving from the anti-polyelectrolyte effect of zwitterionic polymers.

Currently, two main design strategies can be applied to construct temperature-resistant and salt-tolerant acrylamide-based copolymers: introducing (i) zwitterionic structures and (ii) hydrophobic moieties [24–28]. For instance, Du et al. [29] prepared hydrophobically associative polyacrylamide based on p-sulfocalix[4]arene that special structure endows the copolymers with thickening ability, salt tolerance, temperature resistance, and long-term stability. Moreover, Peng et al. [30] synthesized hydrophobically associative polyacrylamide functionalized with β-cyclodextrin. The experiment results illustrated that the copolymers could increase the solution viscosity compared with unfunctionalized polyacrylamides. However, those reported sophisticated techniques and high costs severely limited the further applications in oil recovery. Researchers also prepared composite material by introducing nanoparticles to enhance the thermal-resistant property and reduce costs. Cao et al. [31] prepared amino-functional nanosilica (ANS) by 3-aminopropyltriethoxysilane that improved the viscosity and thermal-resistant ability of polyacrylamide solution on account of ANS showed stronger interaction with functional groups of polyacrylamide. The shortcoming of these composite polymers was poor high salt-resistant property because the nanoparticles were not salt-tolerant enough.

In this work, we designed and fabricated an acrylamide-based copolymer with temperature-resistant and salt-tolerant properties by utilizing the advantages of zwitterions and hydrophobic monomers. First, the amphoteric polyacrylamides consisting of methacryloxyethyltrimethyl ammonium chloride (METAC), acrylic acid (AA) and acrylamide (AM) moieties was synthesized by aqueous dispersion polymerization. This strategy greatly avoids the tedious technology and environmental pollution. The copolymers were characterized by ^1^H-NMR, FT-IR, TG and SEM to confirm that they were prepared successfully and exhibited excellent salt resistant property. The effects of reactants concentrations (e.g., salts, stabilizers and initiators) on the solution viscosity and copolymer size were systematically investigated. Furthermore, different hydrophobic monomers were introduced into amphoteric polyacrylamide chains to improve the thermal-tolerant property. This design strategy is expected to offer a structural direction of the construction for acrylamide-based polymers with improving temperature and salt tolerance via a simple and green technology, presenting both theoretical and practical value for improving the efficiency of oil recovery in high-salinity reservoirs.

## Experimental section

### Materials

Acrylamide (AM,>99%) and acrylic acid (AA,>99.7%) were purchased from Aladdin Co. Ltd. (Shanghai, China). Polyvinyl pyrrolidone (PVP, molecular weight 58,000, K29-32), methacryloxyethyltrimethyl ammonium chloride (METAC), 2,20-azobis (2-amidinopropane) dihydrochloride (V-50) and butylmethacrylate (BMA, >99%) were obtained from Macklin Chemistry Co., Ltd. (Shanghai, China) Stearylmethacrylate (SMA, 96%) was purchased from Rhawn Co.Ltd. (Shanghai, China). Ammonium sulfate (AS), sodium bisulfite (NaHSO_3_), sodium chloride (NaCl), calcium chloride (CaCl_2_), and magnesium chloride (MgCl_2_) were obtained from Sinopharm Chemical Reagent Co.Ltd. (Shanghai, China). Deionized water was purified by a Millipore water purification system.

### Preparation of terpolymer dispersion

Appropriate amounts of ammonium sulfate (AS) were dissolved in water by stirring violently in a three-neck 250-mL glass reactor until completely dissolved. Then, the corresponding proportion of acrylamide (AM), acrylic acid (AA), methacryloxyethyltrimethyl ammonium chloride (METAC) and polyvinyl pyrrolidone (PVP) as a stabilizer were added to the flask with keeping stirring. At the meantime, the reaction was degassed by flowing nitrogen to remove oxygen for 30 min and heated to 50 °C. After that, the appropriate proportion of V-50 as an initiator was added to the reaction. Under a nitrogen atmosphere, the polymerization was carried out at 50 °C for 8 h. After 8 h, a fine white dispersion was obtained at room temperature.

### Preparation of quadripolymer dispersion

The polymerization reactions were carried out under the same conditions as above adding extra the appropriate proportion of hydrophobic monomer butylmethacrylate (BMA) or stearylmethacrylate (SMA).

### Characterizations

The terpolymer and quadripolymer dispersion were dissolved in water and then precipitated by ethanol and acetone, the operation of which was repeated several times to remove the inorganic salts and unreacted monomers.

### FT-IR analysis

The chemical composition of terpolymer was characterized by a Fourier-transform infrared spectroscope (FT-IR; Nicolet 6700) with the wavenumber range of 4000–400 cm^−1^, resolution of 4 cm^−1^ and 32 scans.

### ^1^H-NMR analysis

^1^H-NMR spectrum of terpolymer was obtained by 600 MHz AVANCE III NMR spectrometer (Bruker). The D_2_O was used as the solvent.

### Thermogravimetric analysis (TGA)

The weight loss curves were obtained by thermogravimetric analysis (TGA, Q5000-IR) from 25 °C to 600 °C at the heating rate of 10 °C/min under N_2_ atmosphere.

### The size of copolymer analysis

The particle size was characterized by dynamic light scattering (DLS, NanoBrook-Omni) at 25 °C, and each sample was tested for three times to obtain the average particle size. To verify the temperature and salt resistance capacity of copolymer, the temperature was changed from 20 °C to 80 °C and the salt solution would be applied instead of the water. The composition of salt solution was shown in Table 1 using the salinity of Bohai Sea as model.

**Table 1.** The composition of synthetic BoHai brine solution

Inorganic salt concentration (mg/L)						Total concentration
Na^+^	Ca^2+^	Mg^2+^	HCO_3_^−^	SO_4_^2−^	Cl^−^	
−6100	50	230	100	230	6100	12,810

### Scanning electron microscopy (SEM)

The morphologies of terpolymer were measured by scanning electron microscopy (SEM, FEI Nova Nano 450) at different magnification rates.

### Viscosity analysis

The apparent viscosity of the sample was measured by a rotational viscometer (NXS-11A, China). The content of copolymer was 25 wt%.

### Gel permeation chromatography

The copolymers were characterized by Gel permeation chromatography (GPC) analysis on a Waters-Wyatt system (Waters, Wyatt, USA) at 35 °C using THF as a eluent.

## Results and discussions

**Figure 1.**
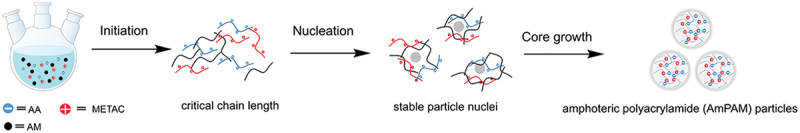
The synthesis procedure of amphoteric polyacrylamide (AmPAM) via an aqueous dispersion polymerization.

**Figure 2.**
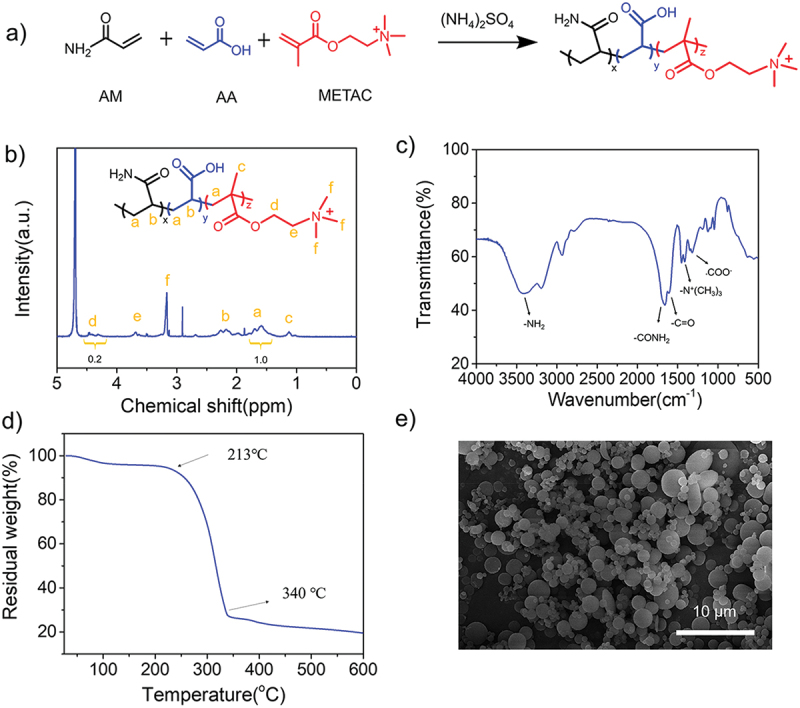
(a) A synthesis route of the amphoteric polyacrylamides (AmPAM). (b) ^1^H-NMR spectrum, (c) FT-IR spectrum, (d) TGA curve, and (e) SEM image of AmPAM.

In this novel dispersion polymerization, as shown in Figure 1, the high concentration of inorganic salt instead of organic solvent induces the phase separation of obtained polymer on account of the salting-out effect. Thus, the monomer, the initiator and the stabilizer are soluble in the initial reaction period, and then the formed polymers gradually separate from the solution at a critical chain length. When the oligomer is formed, it precipitates and becomes stable particle nuclei due to the presence of the stabilizer, and then forms stable polymer particles [32,33]. Compared to the inverse emulsion polymerization, this strategy greatly avoids the tedious technology and environmental pollution [34,35]. As shown in Figure 2a, we first constructed amphoteric polyacrylamide (AmPAM) containing positively charged nitrogen and carboxylate moieties on the polymer backbones. Figure 2b showed the ^1^H-NMR spectrum of AmPAM, indicating that each proton was clearly distinguished from the resonance of the sample. The monomers ratio of the terpolymer (AM: METAC: AA) in the feed was 64:18:18. According to the calculation of the resonance peaks of a (CH_2_ of monomers) and d (-CH_2_-N- of METAC), the composition ratio of terpolymer could be calculated that the molar ratio of (AM and AA) to METAC was approximately 80/20, this result was close to the abovementioned feed ratio. Moreover, FI-IR spectrum in Figure 2c illustrated the composition of the copolymer. The peak at 3403 cm^−1^ was strong and broad, indicating the characteristic peak of amidogen groups. The characteristic signals of 1659 cm^−1^ and 1612 cm^−1^ corresponded to the amide of -CONH_2_ bending vibration band and C = O stretching vibration band, respectively. The signal at 1347 cm^−1^ should be attributed to the stretching vibration band of C-CH_3_, demonstrating the existence of the quaternary ammonium-based groups. In addition, the peak of -COO- was at 1320 cm^−1^ and methyl of -N^+^(CH_3_)_3_ deformation vibration band was 1452 cm^−1^, demonstrating both AA and METAC participated in the dispersion polymerization. The above analysis confirmed the amphoteric polyacrylamide containing positive -N^+^(CH_3_)_3_ group and negative COO^−^ group was synthesized successfully. The thermal stability of copolymer was measured by TGA. As shown in Figure 2d, the weight loss at below 213°C could be attributed to the volatilization of free water and adsorbed water. According to the results of the temperature range of 213–340 °C, a clear decomposition signal of copolymers was detected. It was clearly presented that the terpolymer has a relatively high thermal stability. To observe the intuitive morphology of microspheres, we employed SEM and the SEM image was provided in Figure 2e. It showed that the spherical particles had a uniform surface with an average size of about 2.0 μm.

### The effect of AS concentration

**Figure 3.**
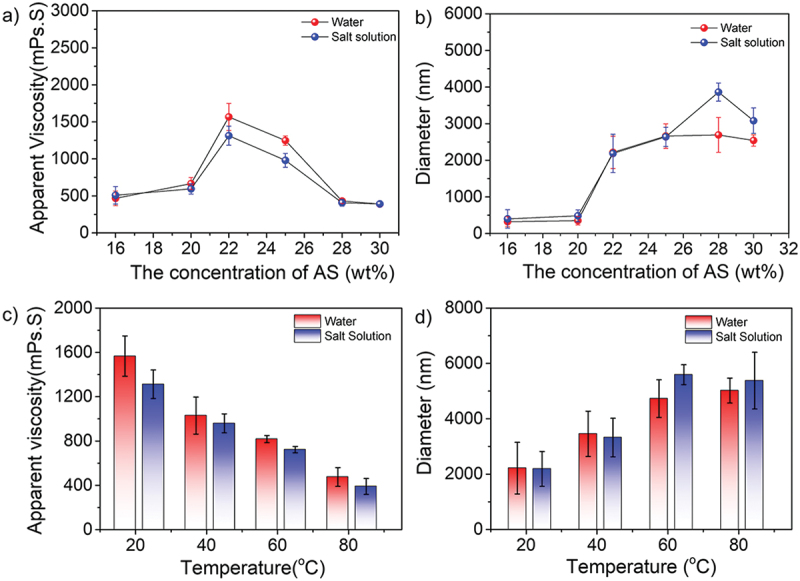
(a-b) Concentration effect of AS (16–30 wt%) and (c-d) temperature-dependent effect (AS concentration: 22 wt%) on apparent viscosity and diameter of amphoteric polyacrylamide particles in water and saline.

To study the effect of ammonium sulfate (AS) concentration in the dispersion polymerization, the different concentrations of AS from 16% to 30% were applied, whereas the concentrations of monomers, stabilizers, and initiators were 15%, 3.0%, and 3.0 × 10^−3^ g/g (monomers)^−1^, respectively. As shown in Figure 3a-b, the apparent viscosity of terpolymer solution increased at first and then decreased as the concentration of AS increased. Significantly, when the concentration of AS was 22%, the corresponding viscosity got to the peak at 1566 and 1012 mPa·s in water and saline (0.0128 kg/L), respectively. Furthermore, it was clear that the diameter of amphoteric polyacrylamide particles increased from 322 to 2886 nm in saline. There are two main reasons: (i) the copolymers could be easily precipitated from saline solution due to the salting-out effect; (ii) increasing salt concentration induced the shrink of the stabilizer molecules, resulting in the effect of stabilization being weakened. Thus, at a lower AS concentration, the dispersed particles were difficult to form with the insufficient salting-out effect and stabilization from PVP. Once the concentration of AS increased and gradually overdose, the critical molecular chain length and nucleation period decreased, and more primary particles were generated. However, the stabilization efficiency of PVP would decrease as the chain of stabilizers shrunk to a larger level, resulting in more particles congregating together. In our system, the proper concentration of AS was 20–28 wt%, which promotes to form the stable copolymer particles. The solution viscosity and particle size of terpolymer were measured at the salinity of 0.0128 kg/L. It was found that the viscosity was slightly lower in the water but the terpolymer size was quite similar even more AS applied, showing a significant salt-resistant property due to the anti-polyelectrolyte effect of the zwitterionic nature. Generally, AS concentration had a certain influence on the apparent viscosity of the copolymer. On the one hand, the viscosity of the system would increase because of a stronger gelation effect, it was harder that monomers and radicals infiltrated the particles due to the high salt-out effect, causing more monomers detained in the continuous phase. Therefore, the viscosity of system was decreased. Under the above-mentioned conditions, the maximum viscosity can be achieved at the AS concentration of 22%. In parallel, the apparent viscosity decreased from 1566 to 475 mPa·s in water and from 1012 to 389 mPa.s in saline with the increasing temperatures, indicating the copolymers have poor temperature-resistant property (Figure 3c). However, the viscosity was quite close in both solutions, demonstrating the good salt-tolerant property. As shown in Figure 3d, the diameter of terpolymer particles increased as the increase of temperature, which can be attributed to the molecular thermal motion was gradually intensified at higher temperatures, and the molecular chains of particles tend to stretch.

### The effect of PVP concentration

**Figure 4.**
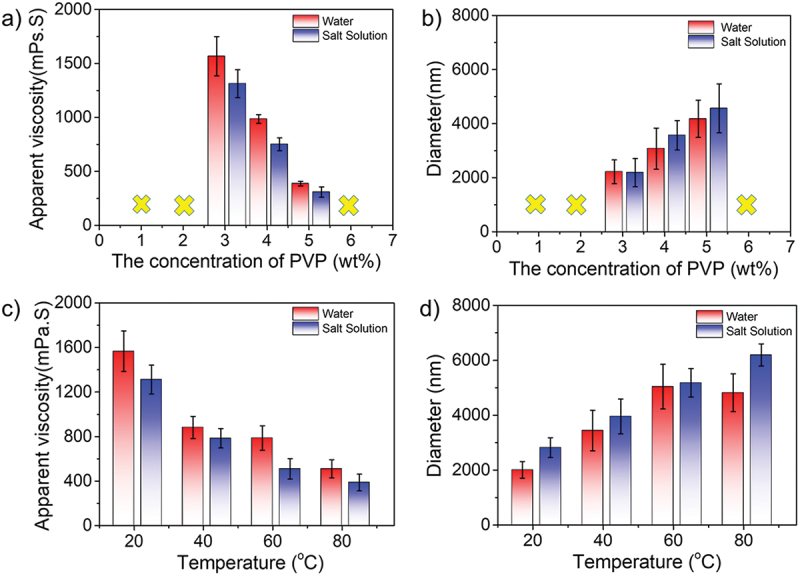
(a–b) Concentration effect of PVP as a stabilizer (3.0–5.0 wt%) and (c–d) temperature-dependent effect (PVP concentration: 3 wt%) on the apparent viscosity (a, c) and particle size (b, d) of amphoteric polyacrylamide in water and saline.

The polyvinyl pyrrolidone (PVP) was selected as a stabilizer in the polymerization experiments, whose concentration was from 1.0% to 6.0%, whereas the concentrations of monomers, salts, and initiators were 15%, 22%, and 3.0 × 10^−3^g/g (monomers)^−1^, respectively. As shown in Figure 4a, when the concentration of PVP was lower than 3.0 wt%, the stabilizer played a poor role in stabilizing particles, causing particles to agglomerate. Similarly, once the concentration of PVP was over 5.0 wt%, some coagulated lumps would appear in the system on account of excess stabilizer molecules tended to connect particles rather than promote stability. It could be seen that the apparent viscosity decreased from 1566 to 386 mPa·s in water with increasing concentration of stabilizers. A similar trend was obtained in saline, as evidenced by the viscosity variation from 1312 to 308 mPa·s. Importantly, the gap was narrow between water and saline, indicating the excellent anti-polyelectrolyte behavior. The change in particle size was shown in Figure 4b, the diameter of particles was gradually increased both in the water and salinity. Specifically, the size of particles was from 2216 to 4175 nm in water and from 2187 to 4563 nm in saline, respectively. In conclusion, the optimum concentration of the stabilizer was 3.0–5.0 wt%. Next, 3.0 wt% PVP concentration was selected for the following temperature-tolerance experiment in Figure 4c,d. It was clear that the apparent viscosity was decreased from 1566 to 510 mPa·s in water and from 1312 to 387 mPa·s when the temperature increased from 20 °C to 80 °C. In the meantime, the particle size in both water and salt solution groups increased with the increasing temperature, which is consistent with our proposed mechanism.

### The effect of initiator concentration

**Figure 5.**
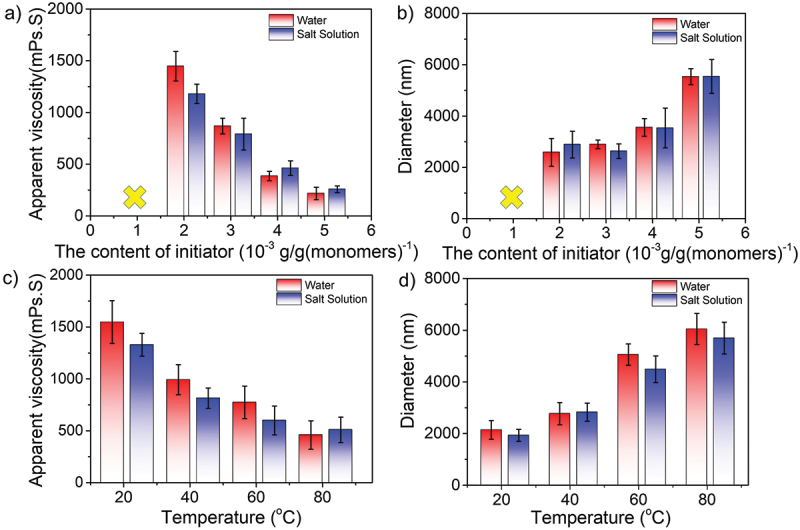
(a–b) Concentration effect of initiators and (c–d) temperature-dependent effect (initiators concentration:  3.0×10^−3^ g/g (monomers)^−1^) on the apparent solution viscosity (a, c) and particle size (b, d) of amphoteric polyacrylamide in water and saline.

As we know, the initiator played an important role in polymerization. Thus, similar synthetic experiments were carried out with the varied concentration of initiator (1.0 ~ 5.0 × 10^−3^ g/g(monomers)^−1^). Other concentration factors were retained as a constant, for instance, 15.0 wt% for monomers, 22.0 wt% for salts, and 3.0 wt% for stabilizers, respectively. Figure 5a-b represented that when the initiator concentration was less than 2.0 × 10^−3^ g/g(monomers)^−1^, excessive unreacted monomers existed in the media, leading to the dispersion being unstable. The results showed the solution viscosity decreased but the particle size increased as the concentration increased of initiators. Statistically, the apparent viscosity varied from 1447 to 216 mPa·s in water and from 1179 to 258 mPa·s in saline, respectively. Such a free-radical polymerization is derived from the high-degree termination of the initiators, thus resulting in the lower average molecular weight of synthetic polymers. On the other hand, the detected particle size increased from 2586 to 5537 nm in water and from 2889 to 5542 nm in saline, mainly because the high concentration of initiator could promote the generation of the unstable primary particles, leading to coagulation and the formation of the larger size particles.

Furthermore, Figure 5c and 5d showed the temperature-dependent (20–80 °C) solution viscosity and particle size variation at the initiator concentration of 3.0 × 10^−3^ g/g (monomers)^−1^. The results were consistent with the abovementioned trends, as the viscosity was decreased, and the particle size increased from 2139 to 6048 nm in water and 1930 to 5696 nm in saline when the temperature increased. The main reason was that an increase in temperature induced the faster motion of the molecular chains, resulting in the intermolecular hydrogen bonds/ electrostatic interactions being destroyed.

### The effect of hydrophobic monomer

**Figure 6.**
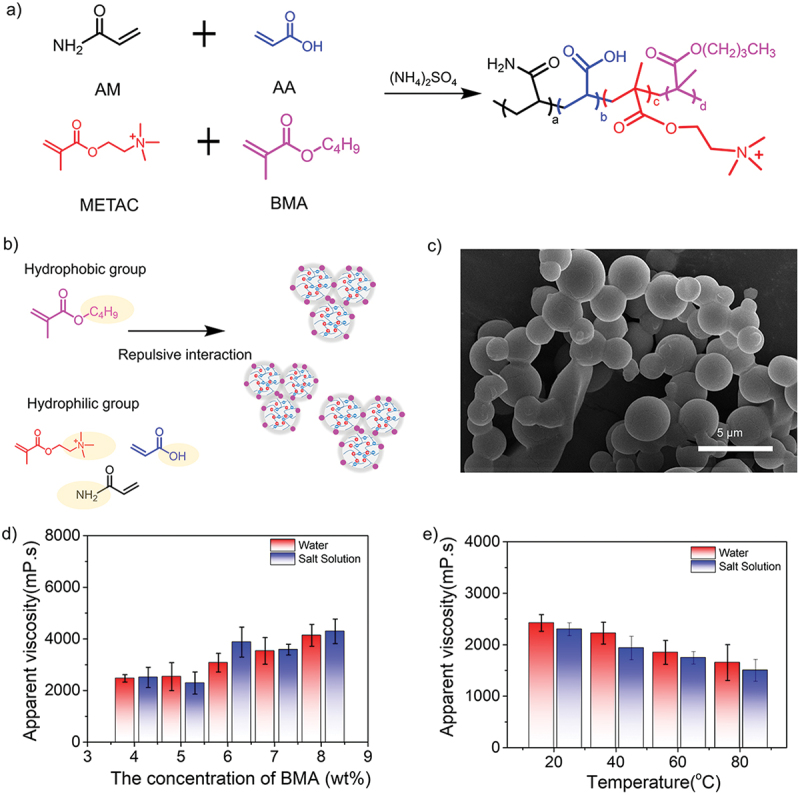
(a) Synthesis routes of acrylamide-based quadripolymers consisting of AM, AA, METAC and BMA. (b) Schematic mechanism of the viscosity optimization for synthetic quadripolymers by introducing the hydrophobic monomers (BMA) with short alkyl chain lengths. (c) SEM image of quadripolymer spheres. (d) Concentration effect of BMA as a hydrophobic monomer (4–8%) and (e) temperature-dependent effect (BMA concentration: 4 wt%) on the apparent solution viscosity of quadripolymer particles in water and saline.

The viscosity of terpolymer solution decreased at high temperatures, significantly influencing the efficiency of reservoir oil displacement. Therefore, we expected that the introduction of hydrophobic monomers would make the resultant quadripolymers exhibit special viscosity properties compared to terpolymer. We further explored the effect of hydrophobic monomers with different alkyl chain lengths (e.g., butyl methacrylate (BMA)) in the dispersion polymerization (Figure 6a). As a hydrophobic monomer with n-butyl moieties, BMA can offer the unique repulsive interaction between hydrophobic and hydrophilic groups along the synthetic copolymers, leading to the obvious thickening property (Figure 6b). Detailed ^1^H-NMR spectrum and FT-IR spectrum were summarized in Figure S1-S2. Specifically, the simultaneous emergence of two new bands at ~1.11 and ~1.18 ppm corresponded to the formation of -CH_2_ and -CH_3_ groups, while the characteristic peaks at 2933 cm^−1^, 852 cm^−1^, and 622 cm^−1^ were assigned to stretching vibrations of -CH_3_, CH_2_- and -(CH_2_)_n_- groups, both results indicating the successful introduction of hydrophobic BMA in quadripolymers. SEM image in Figure 6c presented that the copolymer spheres stuck to each other, indicating the existed unique interactions during the polymerization. We also found that the solution viscosity increased from 2470 to 4837 mPa·s in water and from 2509 to 4892 mPa·s in saline as the BMA concentration increased from 4.0 to 8.0 wt% (Figure 6d). To balance the comprehensive properties, we believe the 4 wt% of BMA is the optimal concentration for the following tests. Similarly, the temperature-dependent effect on the apparent viscosity of quadripolymer particles in the water and saline was investigated in Figure 6e. According to the whole trend, the viscosity tends to become stable with increasing temperatures, showing the introduction of BMA can provide a better temperature-resistant capacity.

**Figure 7.**
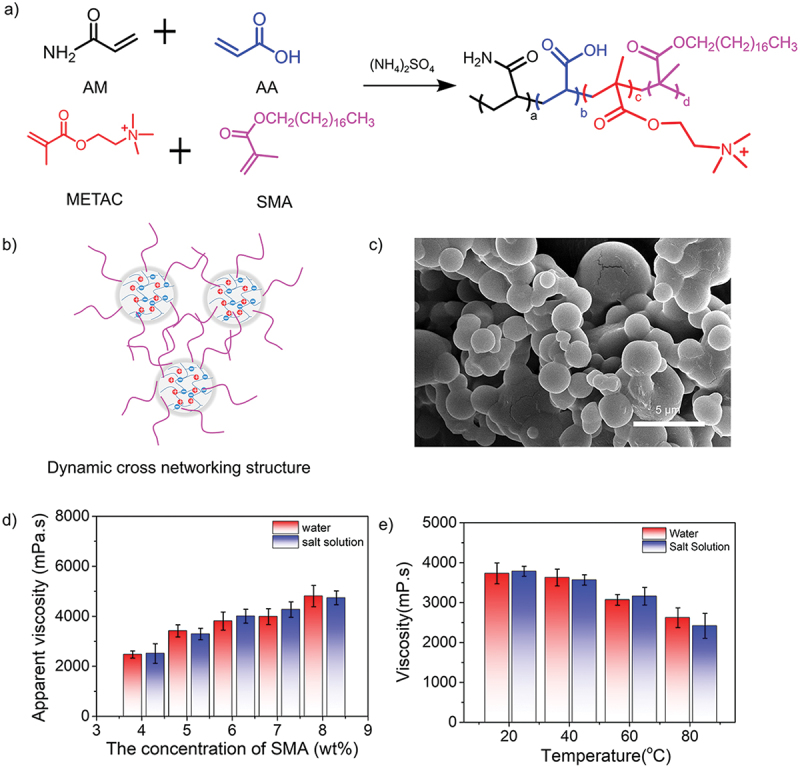
(a) Synthesis routes of acrylamide-based quadripolymers consisting of AM, AA, METAC and SMA. (b) Schematic mechanism of the viscosity optimization for synthetic quadripolymers by introducing the hydrophobic monomers (SMA) with long alkyl chain lengths. (c) SEM image of quadripolymer spheres. (d) Concentration effect of SMA as a hydrophobic monomer (4–8%) and (e) temperature-dependent effect (SMA concentration: 5 wt%) on the apparent solution viscosity of quadripolymer particles in water and saline.

Next, to investigate the effect on the monomers with longer alkyl chain lengths, stearyl methylacrylate (SMA) was selected to prepare new quadripolymers (Figure 7a). Here, the hydrophobic monomers with longer alkyl chains were expected to construct a dynamic crosslinking network to improve the thickening property of the copolymers by hydrophobic association. Since the hydrophobic groups are randomly distributed along the polymer chains, both intermolecular and intramolecular hydrophobic associations may occur in the copolymer solutions (Figure 7b). As shown in Figure S3-S4, the characteristic NMR band appeared at 1.10 ppm corresponding to the -(CH_2_)_n_- group from SMA, while the characteristic peak at 638 cm^−1^ was also attributed to the stretching vibrations of -(CH_2_)_n_- according to the FTIR spectrum. The representative SEM image of synthetic copolymer spheres was provided in Figure 7c. Figure 7d showed that the solution viscosity in both water and saline increased significantly with the increased concentration of SMA. However, as the concentration above the critical association concentration (8 wt%), super-molecular aggregates were formed as the result of the intramolecular association transforming into the intermolecular association, eventually leading to the gelation. Moreover, as shown in Figure 7e, with increasing temperatures, the viscosity retention could be reached 70.2% in the water and 63.8% in the saline, respectively. Moreover, we characterized the molecular weight (MW) of three copolymers using gel permeation chromatography (GPC) in Table S1. The molecular weight quadripolymers were higher than terpolymers, indicating that the more molecular entanglement was achieved, which was corresponding to the high viscosity retention for both quadripolymers. The reason was that the hydrophobic association was deemed to be an endothermic process, therefore, the temperature increase could promote the hydrophobic association. This strategy demonstrated a general working strategy to improve temperature-resistant and salt-tolerant properties.

## Conclusions

In conclusion, we designed and fabricated an acrylamide-based copolymer that possessed both temperature-resistant and salt-tolerant properties by utilizing the advantages of zwitterions and hydrophobic monomers. First, the amphoteric polyacrylamide was synthesized via aqueous dispersion polymerization. The appropriate conditions were as follows: the concentrations of monomers, salts (AS), stabilizers, and initiators were 15 wt%, 20–28 wt%, 3–5 wt%, and 2.0–5.0 × 10^−3^ g/g (monomer)^−1^, respectively. Then different hydrophobic monomers were introduced into amphoteric polyacrylamide to improve the temperature-resistant property. When the short alkyl chain hydrophobic monomer (BMA) was introduced to the terpolymer systems, the viscosity retention of which reached 68.2 % in water and 65.3% in saline at 80°C. Moreover, the viscosity retention could reach 70.2% in water and 63.8% in saline when added SMA with longer alkyl chains. This design strategy is expected to offer a structural direction of the construction for acrylamide-based polymers with improving temperature and salt tolerance, presenting both theoretical and practical value for improving the efficiency of oil recovery in high-salinity reservoirs.

## Supporting information

^1^H-NMR spectra, FI-IR spectra, GPC analysis of terpolymers and quadripolymers.
